# The Bioaccessibility and Antioxidant Activities of Fermented Mango Cultivar Juices after Simulated In Vitro Digestion

**DOI:** 10.3390/foods11172702

**Published:** 2022-09-05

**Authors:** Nobahle P. Cele, Stephen A. Akinola, Tinotenda Shoko, Vimbainashe E. Manhevi, Fabienne Remize, Dharini Sivakumar

**Affiliations:** 1 Phytochemical Food Network Research Group, Department of Crop Sciences, Tshwane University of Technology, Pretoria 0001, South Africa; 2 SPO, University of Montpellier, Universite de La Réunion, INRAE, Montpellier SupAgro, 34000 Montpellier, France

**Keywords:** bioaccessibility, carotenoids, fermented mango juice, lactic acid bacteria, mango cultivar

## Abstract

The purpose of this study was to investigate the bioaccessibilities of total phenolic compounds, carotenoid profile, antioxidant activity, and Lactic acid bacteria (LAB) survival in fermented mango juice (MJs) obtained from three mango cultivars after exposure to an in vitro gastrointestinal digestion model. The MJs from three cultivars (‘Sabre’, ‘Peach’, and ‘Tommy Atkins’) were fermented using *Lactiplantibacillus plantarum* 75 (L75), *Leuconostoc pseudomesenteroides* 56 (L56), and their combination (L56 + 75). Fermented MJs were digested and fractions: gastric (GF), intestinal (IF), and dialysis (DF) were analyzed for total polyphenolic content (TPC), antioxidant activity (FRAP), 1-diphenyl-2-picrylhydrazyl (DPPH), and 2.2-azinobis-3-ethyl-benzothiazoline–6-sulfonic acid (ABTS). In addition, the carotenoid content and the LAB population were determined from the GF and IF. After digestion, TPC decreased while fermentation improved its bioaccessibility. *L75*-fermented ‘Sabre’ MJs had the highest bioaccessible TPC in the GF (75.65%), IF (50.10%), and DF (32.52%) while L56 ‘Peach’ MJs increased the β-carotene bioaccessibility by 1.32-fold at GF and IF (1.21-fold). When compared to the other two juices, ‘Sabre’ and ‘Peach’ MJs fermented with L75 showed the highest IC_50_ values for DPPH and ABTS. Generally, L75-fermented ‘Sabre’ MJs had the highest LAB survival at both GF (7.57 Log CFU/mL) and IF (7.45 Log CFU/mL) and hold potential as probiotic juices. L56-fermented ‘Sabre’ MJs would ensure the delivery of four times the carotenoid recommended dietary allowance (RDA) to a target site in the body while L75-fermented ‘Peach’ MJs could be used to effectively counteract oxidants in the body system.

## 1. Introduction

Among tropical and subtropical fruits, mangoes (*Mangifera indica* L.) are the most common [1]. Several mango cultivars grow globally, among which India alone has over 1500 varieties being the highest producer [2]. Out of those produced in South Africa, the most populous and of economic value include, but are not limited to, ‘Rosa’, ‘Zill’, ‘Irwin’, ‘Peach’, ‘Sensation’, ‘Keitt’, ‘Sabre’, ‘Tommy Atkins’, ‘Phiva’, ‘Haden’, and ‘Kent’ [1,2]. A large proportion of mango production in South Africa is consumed locally as food [3]. Mango juice is a rich source of antioxidants such as ascorbic acid, phenolic compounds, and carotenoids [4]. The antioxidant properties of mango fruits are of great importance when evaluating their quality for market and human health [5]. Aside from the role of carotenoids as antioxidants in preventing some diseases related to oxidative stress, the high level of carotenoids in mango fruit makes it a good choice for treating vitamin A deficiency [5]. Moreover, mango juices hold the potential to meet the Recommended Dietary Allowance (RDA) requirements of carotenoids for different classes of the population [6]. 

Most indigenous crops are underutilized despite their laden potential to contribute to the nutrition and health of the consumers. During bumper harvest, the non-commercial cultivars of mango fruits undergo rot and spoilage due to under-exploitation. Despite mangoes’ nutritional and sensory properties, their processing and utilization remain limited due to inappropriate processing technologies to preserve their quality and utilization after harvest. Therefore, this study seeks to advance the utilization of notable indigenous mango cultivars in South Africa. 

Extensive studies have been conducted on mango fruits and juices; however, limited information exists on the bioactive compounds contained in indigenous South African mango cultivars. Our previous study reported the preservation of ascorbic acid, carotenoids, phenolic compounds, and antioxidants after 24 h fermentation of indigenous mango cultivar juices with select Lactic acid bacteria (LAB) strains [6]. Similarly, Mapelli-Brahm et al. [7] had earlier reported the potential of fermentation in improving the retention and bioaccessibility of the carotenoids in fruits and vegetables, while Wang et al., [8] confirmed this observation in ultrasonic pre-treated fermented mango juice after lactic acid bacteria fermentation. The bioaccessibility of functional compounds in indigenous mango cultivars could help improve the nutrition and health of its consumers. However, the bioaccessibility of the essential nutrients in mango juice relies on its ability to reach the target sites in the body by making it through the harsh digestive process of the gastrointestinal tract. Hence, this study investigated the effect of gastrointestinal (GI) digestion on the phenolic compounds, carotenoids, and antioxidant activities of LAB-fermented mango juices obtained from selected indigenous mango cultivar fruits of South Africa.

Bioactive compounds in juices could function as prebiotics for the survival of probiotics [9]. Probiotics are desirable microorganisms that can modulate the GI tracts and deliver functional benefits to the host. The use of desirable microorganisms such as lactic acid bacteria in foods could be a strategy to access possible health benefits. The inclusion of probiotics in foods provides benefits such as the improved nutritional value of food, control of intestinal infections, improved digestion of lactose, control of some types of cancer, modification of sensory attributes, improved antioxidant levels, and biotransformation by metabolizing of phenolic compounds [10,11]. 

Microorganisms belonging to the genera of Lactobacillus and Bifidobacteria are the most used probiotics, and the efficacy of their biological activities are strain-dependent [12]. *Lactiplantibacillus plantarum* (*Ltp. plantarum*) is a known versatile strain of lactic acid bacteria belonging to the *Lactobacilli* genus that is often used in the fermentation of foods due to its ability to survive in harsh conditions, and its potential to exhibit probiotic properties [10,13]. *Ltp. plantarum* strains are adapted to the human mucosal environment and other ecological niches [13]. Similarly, *Leuconostoc pseudomesenteroides* (*Leu pseudomesenteroides*) are lactic acid bacteria belonging to the *Leuconostoc* genera which have been identified for their antimicrobial activities. In addition to this, it has been classified and used as probiotics in food, based on its ability to survive conditions in the GI tract [10,14]. Hence, the choice of these strains in the fermentation of mango juices in this study.

The determination of bioactive compounds in foodstuff directly is not enough for the prediction of its potential effects in vivo. The capability of a bioactive compound (hydrophilic or lipophilic) to exert a health benefit is dependent on its ability to withstand food processing, release from the food matrix post-ingestion, bioaccessibility in the gastrointestinal tract, and ability to reach the target tissue of action [15]. It is generally accepted that in vitro digestion of foods is an effective tool for assessing the bioaccessibility of compounds in foods in relation to their biological activity. However, in vivo and clinical placebo trial studies are time-consuming, costly, and have ethical restrictions [10], hence, in vitro models offer an alternative for the investigation of the fate of bioactive compounds in LAB-fermented mango juice obtained from different cultivars after digestion. The in vitro models have been employed to predict the release of compounds in complex food matrices, changes in bioaccessibility, and metabolite profiles of MJs [16]. However, none has reported the effect of digestion on the fate of bioactive compounds in LAB-fermented MJs from different indigenous cultivars in South Africa. Therefore, this study investigated the fate and bioaccessibilities of total phenolic compound, carotenoid components, antioxidant activities, and LAB survival of LAB-fermented MJs obtained from different cultivars after exposure to simulated gastrointestinal digestion.

## 2. Materials and Methods

### 2.1. Chemicals

All chemicals and other reagents were purchased from Sigma Aldrich, Johannesburg, South Africa. *Leuconostoc pseudomesenteroides 56* (*L56*) and *Lactiplantibacillus plantarum 75* (*L75*) were obtained from the Microbiology Laboratory at QualiSud, Université de La Réunion for this study, Réunion, France.

### 2.2. Mango Juice Preparation

Mango cultivars ‘Tommy Atkins’, ‘Peach’, and ‘Sabre’ were harvested from the orchards in Venda, Limpopo, South Africa at the commercial ripe stage (stage 4). Preparation of mango juices for fermentation with LAB strains was performed according to Cele et al. [7]. A sterile knife was used to remove the peel of mango fruits after they were thoroughly washed in tap water. A Kenwood juicer (Havant, UK) was used to extract mango pulp, and mango pulp was diluted with sterile portable water at a ratio of 1:2 (pulp: water; *v*/*v*). For inoculation, the juice was pasteurized at 80 °C for 15 min in a water bath and cooled to room temperature. The LAB cultures: *L75* and *L56* were reactivated in MRS broth medium (Sigma Aldrich, St. Louis, MO, USA) for 48 h at 30 °C. LAB cells were cultured aerobically and harvested by centrifuging for 5 min at 8000× *g*. The cells were washed in sterile water and were later suspended in phosphate-buffered saline solution. The cell population was determined at 660 nm using a UV- spectrophotometer (SpectroStar Nano, BMG LABTECH, Ortenberg, Germany). An equal volume of *L75* and *L56* harvested cells (1/1: *v*/*v*) was used as the combination treatment (*L56 + 75*). Portions from each LAB cell culture were mixed in an Erlenmeyer flask and the cell population was adjusted to 8 Log CFU/mL prior to the juice inoculation. One milliliter of 8 Log CFU/mL of LAB culture was inoculated into 19 mL of juice and allowed to ferment for 24 h at 37 °C. The unfermented mango juice from individual cultivars served as the controls.

### 2.3. In Vitro Digestion of Fermented MJs

**Figure 1:**
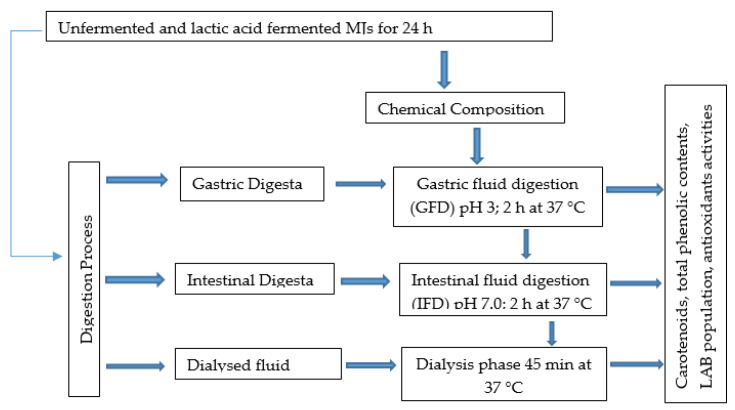
Schematic diagram of simulated in vitro GI digestion of unfermented and fermented MJs incubated for 24 h at 37 °C.

Following the Infogest nature protocols, as described by Managa et al. [10], fermented mango juices were subjected to simulated gastrointestinal digestion. As presented in Figure 1, all fermented MJs were incubated at 37 °C for 24 h and were subjected to successive gastric and pancreatic conditions. Briefly, 10 mL of homogenized fermented MJs was mixed with 15 mL of simulated gastric fluid (SGF) to get 40 mL of the final volume. The pH was adjusted to 3 with 0.1 mL HCl solution and 10 mL of pepsin (2000 U/mL) was added. Under agitation, the mixture was incubated for 2 h at 37 °C. The test tubes were then cooled on ice to stop the reactions and aliquots were taken for later analysis. The intestinal digestion phase was performed in two successive steps: the agitation step and the dialysis process. Fermented mango juice at the gastric phase was mixed with simulated intestinal fluid (SIF) to 80 mL final volume after the addition of pancreatin (100 U/mL) and bile salt (10 mM) and the pH was adjusted to 7 and was then incubated for 2 h at 37 °C. The intestinal phase was followed by the collection of aliquots for analysis. The dialysis was performed in a tubing cellulose membrane (MWCO 10 kDa) to make a simplified model of the epithelial barrier. Dialysis bags filled with 5.5 mL NaCl (0.9%) and 5.5 mL NaHCO_3_ (0.5 M) sealed with clips were completely immersed into the GI digesta immediately after digestion. The fermented juice-digested fractions in dialysis bags were incubated for 45 min at 37 °C under agitation. Aliquots were collected for analysis at the end of the incubation time.

### 2.4. Effect of In Vitro Digestion on the Survival of LAB in Fermented MJs

The surviving LAB population in digested fermented MJs obtained from different cultivars was determined according to the method described by Managa et al. [10]. An aliquot of digested fraction derived from fermented MJs from different cultivars was ten-fold diluted serially in sterile saline water (0.85%). On MRS agar, appropriate dilutions were plated and incubated at 37 °C for 48 h at 30 °C. After each digestion phase, the surviving LAB population was enumerated and expressed as Log CFU/mL of MJs digest. 

### 2.5. Total Phenolic Content

As described by Cele et al. [6], the Folin–Ciocalteu assay was used to determine the total phenolic content of the MJs. A 200-µL aliquot of digested fraction was mixed with a 1000 µL diluted Folin–Ciocalteu reagent. Thereafter, 800 μL of 7.5% Na_2_CO_3_ solution was added and the mixture was incubated at room temperature for 2 h. The absorbance was measured at 750 nm in a spectrophotometer (SPECTROstar Nano, BMG LABTECH, Ortenberg, Germany) blanked with distilled water. The TPC concentration was then calculated from a calibration curve, prepared with 1 μM gallic acid. 

### 2.6. Carotenoid Profile of MJs after Digestion

Carotenoid profiles of MJs digests (20 mL) in 150 mL tightly sealed tubes were extracted with acetone: hexane (1:1) containing 0.1% BHT incubated overnight in the dark as described by Moloto et al. [17] and Cele et al. [6]. The residue was rinsed with 5 mL volumes of the extraction solvent and centrifuged (Model Hermle Z326k, 153 Hermle Labortechnik, Wehingen, Germany) at 3000× *g* for 5 min at 5 °C. Supernatants were dried in anhydrous sodium sulphate, filtered with Whatman filter paper (No 1), and evaporated to dryness under nitrogen gas. Extracts were re-dissolved in isopropyl alcohol (10%) and n- hexane and then filtered through a 0.45 µm PTFE syringe filter before quantification in a High-Performance Liquid Chromatography (HPLC). The HPLC Shimadzu Prominence-I LC-2030C 3D Liquid chromatograph equipped with an LC-2030 autosampler and LC-2020/2040 Photodiode-Array 160 Detection detector (Kyoto, Japan) was used. A 250 mm × 4.6 mm id. 5 µm Shim pack GIST NH2 column was used in detection at a wavelength of 460 nm at 30 °C and injection volume of 10 µL and a flow rate of 0.6 mL/min. A pure standard was used to identify β-carotene, zeaxanthin, trans-beta carotene, alpha-carotene, and lutein absorption peaks in digests. Based on the standard curve, the least of determination (LOD) was 13.82 and the least of quantification (LOQ) was 46.06 g/mL. The quantification and validation of the carotenoid contents were obtained from the calibration curve as shown in the Supplementary Materials (Table S1). 

### 2.7. Antioxidant Activities of Fermented MJs Digest

#### 2.7.1. Ferric Reducing Antioxidant Power (FRAP)

Antioxidant power was determined by combining 10 mmol/L TPTZ (2,4,6-tris(2-pyridyl)-1,3,5-triazine) dissolved in 40 mM HCl with 20 mM FeCl_3_ in a 1:1:10 ratio having 20 mM acetate buffer (pH 3.6) [6]. A 0.1 g of freeze-dried MJs digest was reconstituted in 80% methanol, and to 20 µL of the digest, 150 µL FRAP reagent was added in a microtitre plate and incubated for 10 min. A microplate reader (SPECTROstar Nano, BMG LABTECH, Ortenberg, Germany) was then used to measure the absorbance at 593 nm. As a reference standard, Trolox solutions from 0 to 30 mM were prepared and the results were expressed in mM TEAC/g.

#### 2.7.2. 1,1-Diphenyl-2-picryl-hydrazyl (DPPH) of Fermented MJs Digest 

DPPH radical scavenging antioxidants were determined as described by Cele et al. [6]. An aliquot of 0.1 mM DPPH solution was prepared in methanol (3 mL) at different concentrations of the digest (0.5–15.0 mg) dissolved in 150 μL methanol. The mixture was transferred into a microtitre plate and incubated for 30 min in the dark. The absorbance of the mixture at 593 nm was measured using a microplate reader (SPECTROstar Nano, BMG LABTECH, Ortenberg, Germany) with reference to the Trolox standard (0-200 µg/mL). The antioxidant concentration of digest needed to reduce DPPH absorbance by 50% (IC_50_) was calculated and expressed as IC_50_ (mg/mL) of MJs digest. 

#### 2.7.3. 2,2-Azinobis-(3-ethyl-benzothiazoline-6-sulfonic acid (ABTS) of Fermented MJs Digest 

The ABTS radical cation (ABTS+) was made by mixing 7 mM ABTS stock solution with 4.9 mM potassium persulphate in a 1:1 ratio and storing the solution at 25 °C for 12 to 16 h before use according to Cele et al. [6]. To 1500 μL of ABTS+ solution, varied concentrations of MJs digest (20, 70, 80, and 100 μL) were added. A UV-spectrophotometer (SPECTROstar Nano, BMG LABTECH, Ortenberg, Germany) measured the decline of absorbance after adding 40 μL of the digest to the ABTS+ solution. The antioxidant concentration needed to reduce ABTS+ absorbance by 50% (IC_50_) was expressed as IC_50_ (mg/mL) [6].

### 2.8. Statistical Analysis

One-way analysis of variance was used to analyze the effect of digestion on surviving LAB population, antioxidants, and carotenoid concentrations using the statistical software (GenStat version 11.1, Hemel Hempstead, UK). The means were separated using Fisher’s least significant difference at a 5% confidence interval, and results were presented as mean ±SD. For each parameter, five replicates were conducted.

## 3. Results and Discussion

### 3.1. LAB Surviving Population in Fermented MJs after In Vitro GI Digestion

**Figure 2:**
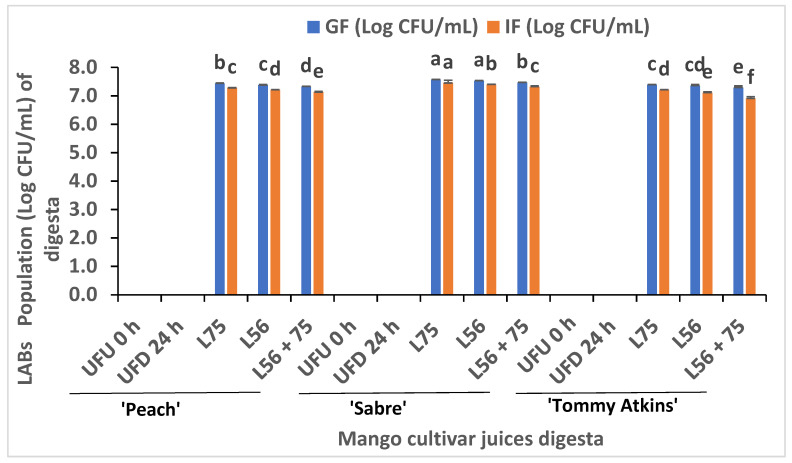
Survival of LAB from fermented MJs of three different cultivars during in vitro GI digestion. Similar colored bars with the same alphabetic letter are not significantly different (*p* ≤ 0.05). Keys: UFU 0 h—raw unfermented and undigested MJs (control); UFD 24 h—unfermented digested MJs stored for 24 h; *Ltp. plantarum* (L75); *Leu. pseudomesenteroides* (L56); *Leu. pseudomesenteroides* 56 + *Ltp. plantarum* 75 (L56 + 75); gastric fraction (GF); Intestinal fraction (IF), Log CFU/mL = Logarithmic colony forming unit per milliliter of digested fractions.

The effect of digestion on the LAB survival of fermented and unfermented MJs is presented in Figure 2. The phase of digestion, LAB strains involved in fermentation and mango cultivar type affected the population of surviving LAB. The LAB population in all three mango cultivar juices fermented by either *L75*, *L56*, or its combination after gastric phase digestion ranged from 7.29 to 7.57 Log CFU/mL. ‘Sabre’ MJs fermented with *L75* (7.57 Log CFU/mL) and *L56* (7.53 Log CFU/mL) showed the highest LAB survival followed by L56 + 75 (7.29 Log CFU/mL). Whilst the GF of *L75*-fermented ‘Peach’ and ‘Tommy Atkins’ MJs digesta’s had 7.44 Log CFU/mL and 7.39 Log CFU/mL LAB population, respectively. There were no significant differences between the LAB populations in *L75* and *L56*-fermented MJs at the GF (*p* < 0.05) except for the ‘Peach’ cultivar. Although the survival of L56 + 75 in all three MJs was significantly lower than *L75* and *L56*, ‘Sabre’ MJs showed the highest (7.47 Log CFU/mL) and the lowest survival in L56 + 75-fermented ‘Tommy Atkins’ gastric fraction (7.29 Log CFU/mL). 

Moreover, at the intestinal phase, the *L75*-fermented ‘Sabre’ MJs showed the highest cell population (7.45 Log CFU/mL), followed by ‘Peach’ (7.28 Log CFU/mL) and ‘Tommy Atkins’ MJs. The LAB population decreased slightly as the acid-based environment changed during the transition from the stomach to intestinal phase, while the cells of L56 + 75 from the ‘Tommy Atkins’ MJs decreased from 7.29 Log CFU/mL to 6.91 Log CFU/mL, which could be attributed to the higher pH (alkaline) condition. On the other hand, the presence of fructose in the MJs may have aided the survival of lactic acid bacteria. According to Haveenar and In’t Veld [18], probiotics are viable microorganisms that exist either as mixed or monocultures and are able to improve the health of the host through the improvement of gut microflora balance and the modulation of dysbiosis. The ability of a probiotic strain to survive passage through the acidic gastric environment and to attach to the epithelium cells in the intestine or to the mucus layer will greatly affect the stability and multiplication of probiotic bacteria in the intestine. Probiotics must be alive and plentiful after ingestion to be able to provide a probiotic function according to the FAO/WHO proposed definition [19]. According to research reports, the minimum doses of 10^6^–10^9^ alive probiotic cells per day in the gut are needed to exert beneficial effects [20]. On this basis, the *Lactobacillus* and *Leuconostoc* strains: L75, L56, and L75 + 56 demonstrated cell survival of greater than 10^6^ cells. The use of metabolites such as phenolic compounds by a viable organism in the gut may also qualify it as an active microorganism [19]. Furthermore, a true probiotic must be viable and active more than its survival. According to Kumar and Singh [21], probiotic strains have health benefits in the gut; therefore, future research should examine whether they are true, pseudo, or ghost probiotics and whether they benefit human health.

### 3.2. Changes in TPC of LAB Strains Fermented MJs from Different Cultivars after In Vitro GI Digestion

A growing body of evidence suggests that phenolic compounds in foods are beneficial to human health [22]. Some food phenolic compounds are transformed by fermentative microbiota during fermentation. This transformation is frequently required for absorption and regulation of the biological activity of dietary compounds. It has been shown by Selma et al. [23] that the bioavailability and effects of phenolic compounds are greatly dependent on their transformation by specific fermentative microbes utilizing esterases, glucosidases, dehydroxylases, and decarboxylases. Fermentation has an optimistic effect on overall phenolic content; however, the magnitude of this influence is reliant on the microorganism involved. 

Moreover, the effect of digestion on the phenolic components, fold increase, and bioaccessibility of fermented and non-fermented MJs at the GF, IF, and DF is as presented in Table 1. The TPC of fermented MJs of all three cultivars (‘Peach’, ‘Sabre’, and ‘Tommy Atkins’) decreased in GF when compared to the UFU 0 h and its contents ranged from 372.19 mg/100 mL to 1171.41 mg/100 mL. Conversely, the TPC in all MJs from all three cultivars significantly decreased in digested fractions of fermented (L75, or L56 or L56 + 75) and unfermented MJs fractions (UFD 24 h) when compared to their respective UFU 0 h. The higher TPC concentration in the GF of fermented MJs compared to the unfermented samples confirms the effect of fermentation on the increase of TPC in MJs fermented by L75, L56, or L56 + 75 as previously reported by Cele et al. [6]. In the *L75*-fermented MJs, GF had the lowest fold decrease in TPC compared to the MJs fermented with *L56* or L56 + 75 and was lowest in ‘Sabre’ MJs (1.23-fold) followed by ‘Peach’ (1.42-fold) and ‘Tommy Atkins’ (1.86-fold). Similarly, the L75-fermented ‘Sabre’ (81.16%), ‘Peach’ (70.19%), and ‘Tommy Atkins’ (53.74%) MJs digests had the highest bioaccessible TPC compared to L56 or L56 + 75 and unfermented MJs at the GF. In comparison to MJs from all three cultivars, ‘Sabre’ MJs fermented with L75 showed the highest retention of TPC in the GF.

Intestinal membranes of the upper gastrointestinal tract facilitate the absorption of phenolic compounds [24]. The TPC decreased from GF to IF phase, a further decrease occurred in the DF of all fermented MJs compared to their respective unfermented MJs. Compared to all UFD MJs, ‘Tommy Atkins’ 24 h MJs had the lowest TPC concentration in the IF and DF at 241.77 mg/100 mL and 172.95 mg/100 mL, respectively. Those fermented with L75 had higher retention of TPC by demonstrating a lower fold decrease than the L56 or L75 + 56 in both the IF and DF. Conversely, L75-fermented ‘Sabre’ MJs demonstrated the highest retention of bioaccessible TPC in the respective IF and DF (55.61% and 34.23%) than ‘Peach’ (43.66% and 31.26%) or ‘Tommy Atkins’ (35.71% and 30.68%) MJs fractions. In MJs, fermentation enhances the release of TPC. A similar result was reported for LAB-fermented kiwi fruit [25]. Also, this observation was in agreement with the increase of total polyphenolic compounds in *Ltp. plantarum*-fermented nightshade leaves which hydrolyzed flavonoid conjugates during fermentation, thereby increasing the polyphenol bioavailability [26].

Furthermore, the stability of the phenolic compounds might be associated with a lower pH caused during fermentation and the consequent production of lactic acid [25]. The increased bioaccessible TPC in digested fractions might be due to a continuous breakdown of phenolic compounds by the fermenting LAB strains during gastrointestinal digestion, thus implying continuous metabolic activity in the gastrointestinal tract. According to this observation, sugar depletion prompted the bacteria to utilize phenolic compounds, consequently increasing bioaccessibility through biotransformation. This observation agrees with the higher population of surviving LAB after digestion. The Folin–Ciocalteu method used in this study was able to detect the bound compounds that were free after pepsin hydrolysis during digestion. Moreover, the observed increase in TPC bioaccessibility could be explained by the synergistic effect of the original phenolic compounds released from fruit juice combined with the production of new compounds in acidic conditions, or by oxidation, as suggested by Succi et al. [11]. This is consistent with the findings of Rodríguez-Roque et al. [27] on the increased bioaccessibility of polyphenols after in vitro gastric digestion. The observed decrease in TPC at the IF and DF phases can be attributed to the higher alkaline pH indicating that the majority of phenols are sensitive to alkaline conditions (pH = 7.2). DF is defined as the fraction that is capable of being absorbed by diffusion into the systemic circulation [28]. Furthermore, the observed decrease in TPC and survival of the cells of L56 + 75 in the IF phase (Figure 2 and Table 1) during the digestion of MJs from across all three cultivars showed that the transition from acid pH to alkaline pH had affected the biotransformation of phenolic compounds.

### 3.3. Changes in Carotenoids of MJs from Different Cultivars Fermented with LAB after In Vitro GI Digestion

The bioavailability and solubilization of carotenoids in the digestive tract determine their biological activity [29]. Several factors influence the absorption of carotenoids, including the matrix of the food and the type of carotenoid [30]. The effect of GI digestion on the carotenoid contents of fermented and unfermented MJs is shown in Table 2. The β-carotene content of fermented MJs from different cultivars after digestion ranged from 14.4 to 80.63 mg/mL in the GF and IF (12.63–50.43 mg/mL). Surprisingly, five types of carotenoids were detected after digestion of MJs as shown in Table 2. In the GF, the trans-β-carotene and zeaxanthin concentrations of fermented MJs from different cultivars ranged from 3.03 to 9.17 g/mL and 2.06 to 8.16 g/mL, respectively. Furthermore, the lutein concentration of LAB-fermented MJs after digestion ranged from 2.4 to 6.5 µg/mL at the GF and 0.6 to 6.16 µg/mL at the IF while the α-carotene content in fermented MJs after digestion at the GF ranged from 3.36 to 7.5 µg/mL and 1.26 to 5.5 µg/mL at the IF (Table 2). β-carotene, trans-β-carotene, zeaxanthin, lutein, and α-carotene levels increased significantly in all fermented MJs digested fractions across cultivars at the gastric phase (*p* ≤ 0.05), compared to their respective unfermented and undigested MJs with higher concentration in L56-fermented ‘Sabre’ MJs and a significant 2.59-fold increase. In the gastric fraction, β-carotene decreased in the LAB-fermented and unfermented (UFD 24 h) ‘Tommy Atkins’ MJs compared to ‘Peach’ and ‘Sabre’ cultivars that had an increase in β-carotene content except in the L56-fermented ‘Tommy Atkins’ MJs that had a 1.27-fold increase. The quantity of the bioaccessible carotenoid in the GF increased with the digestion of fermented MJs, and its values ranged from 71.29% in ‘Tommy Atkins’ (UFD 24 h) to 258.94% in L56-fermented ‘Sabre’ MJs which had the highest bioaccessible β-carotene (Table 2). The increased bioaccessibility could be due to the depletion of triglycerides in MJs during fermentation which enabled an increased micellarization and absorption of non-polar carotenoids in the digest [31]. 

Similarly, there was a significant increase in bioaccessible trans-β-carotene and zeaxanthin contents (*p* ≤ 0.005) in *L56*-fermented MJs: ‘Tommy Atkins’ (2.03-fold, 203.63%; 2.5-fold, 250.48%), ‘Sabre’ (1.78-fold, 178%; 1.99-fold, 199%), and ‘Peach’ (1.74-fold, 174%; 1.91-fold, 191.5%) at the GF (Table 2) compared to a decrease at the intestinal phase. However, a slight increase in bioaccessible β-carotene, trans-β-carotene, and zeaxanthin in unfermented and fermented MJs digest fractions of ‘Peach’, ‘Tommy Atkins’, and ‘Sabre’ cultivars at the gastric phase could be attributed to the depolymerisation/release of complex polysaccharides such as pectins in the MJs. Consequently, there was a drastic reduction of the β-carotene in the IF of digested MJs. The fold decrease in β-carotene of the IF was greatest in the UFD 24 h ‘Tommy Atkins’ MJs (1.60-fold decrease) indicating a rapid degradation of the carotenoids in L75, and L56 + 75-fermented and digested MJs. Conversely, ‘Sabre’ MJs fermented with *L56* had a significantly lower relative fold decrease in β-carotene (1.01-fold), thus signifying the preservation of β-carotene at the intestinal phase, compared to the other fermented MJs of ‘Peach’ and ‘Tommy Atkins’ cultivars and their respective UFD 24 h MJs digest fractions. However, the intestinal fraction of *L56* fermented ‘Sabre’ MJs (98.99%) had the highest bioaccessible β-carotene compared to their counterpart of ‘Tommy Atkins’ (90.84%) and ‘Peach’ (82.40%).

Furthermore, there were significant increases in lutein concentration of fermented MJs at the GF and was highest in L56-fermented MJs compared to UFD 24 h and other fermented MJs from different cultivars. The bioaccessible lutein content was lowest in UFD 24 h ‘Tommy Atkins’ MJs (108.3%) while L56-fermented and digested ‘Sabre’ and ‘Tommy Atkins’ MJs had the highest bioaccessible lutein content (187%) and were not significantly different from each other (*p* ≤ 0.05). The enzymatic activities of lactic acid bacteria colonising the gut have been reported to enhance the bioaccessibility of carotenoids, thus, the observation in this study might be due to the enzymatic hydrolysing abilities of lipase of active LAB strains in fermented MJs [10]. The low-fat content in MJs caused by the effect of fermentation might have aided the increased micellarization and bioaccessibility of lutein in MJs digest [30,31].

Similar to the observation in β-carotene content, the bioaccessible trans-β-carotene, zeaxanthin, and lutein content decreased in MJs at the IF stage except in L56 and L56 + 75-fermented MJs and were highest in L56-fermented ‘Peach’ MJs compared to other *L75* and L56 + 75-fermented, unfermented, and undigested MJs. A significant fold increase in bioaccessible trans-β-carotene and zeaxanthin was observed in L56-fermented intestinal fractions (*p* ≤ 0.005). However, there were no significant differences between the bioaccessible trans-β-carotene and zeaxanthin contents of L56-fermented ‘Peach’ and ‘Tommy Atkins’ MJs signifying the potential of L56 to preserve carotenoids in MJs, thus supporting the previous observation of Cele et al. [6] in fermented MJs. Compared to other LAB-fermented, unfermented, or undigested MJs, L56-fermented ‘Sabre’ MJs had the highest bioaccessible lutein (156.74%) in IF, thus supporting the observation of increased bioaccessibility of less polar lutein in digested spinach compared to β-carotene [31]. Hence, the digestion and the acidic condition of fermented MJs would have aided the release and recovery of lutein in MJs.

Moreover, there was an appreciable fold increase in α-carotene contents in the fermented and digested MJs observed at the GF and a decrease at the IF similar to the observation in other assayed carotenoid profiles. Similarly, L56-fermented MJs had a higher concentration of α- carotene than other LAB-fermented and unfermented MJs after digestion, similar to what was observed for zeaxanthin, lutein, and trans-β-carotene. Digestion of fermented MJs from the ‘Tommy Atkins’ cultivar aids the release of bound α- carotenoids when fermented with the L56 strain. High fold increase and bioaccessible α-carotenoids were observed in L56-fermented ‘Tommy Atkins’ MJs (1.63-fold, 163.69%) at the GF while at IF, the bioaccessible α-carotene was significantly lost in the fermented and digested MJs, thus supporting the report that the liberation of carotenoids is a function of the food matrix, pH, triglycerides, and fatty acid composition of food [31]. ‘Tommy Atkins’ MJs might have possessed some unique triglycerides that enhanced the liberation of bioaccessible carotenoids in this study. Similarly, the change from acidic to basic pH might have a depleting effect on α- carotenoid content on LAB-fermented MJs except in L56-fermented ‘Peach’ (111.5%) and ‘Sabre’ (113.15%). 

The higher β-carotene in ‘Peach’ cultivar MJs fermented by L56 could possibly be due to its unique food matrix [32] taking into account its fibreless nature, however, the acidic pH at the gastric phase aided the solubilisation of the carotenoid in the MJs. The significant decline in bioaccessible β-carotene, trans-β-carotene, zeaxanthin, lutein, and α- carotene at IF compared to GF might be due to a rise in the pH thereby reducing the stability of the carotenoids. The higher concentration of bioaccessible carotenoid profiles (trans-β-carotene, zeaxanthin, lutein, and α-carotene) in the L56-fermented MJs from ‘Tommy Atkins’ cultivar after GF digestion compared to other cultivars of fermented MJs could be due to the ease of biotransformation of the carotenoids based on a unique dietary component in the fruit. As a result of the low sugar content in ‘Tommy Atkins’ MJs, the fermenting LAB may have been able to biotransform more carotenoids. The higher concentration of carotenoids in L56-fermented MJs digest could be related to the ability of these probiotics to produce some short-chain fatty acids which improve the biotransformation of carotenoids. Saxholt et al. [33] have previously reported the enhanced β-carotene liberation due to the presence of the short-chain or long-chain fatty acids in foods. Therefore, the mango cultivar and LAB type influence the recovery and bioaccessibility of carotenoids in MJs. Additionally, mango’s high plastoglobule content may have enhanced carotenoid bioaccessibility after digestion [34]. Fermented ‘Tommy Atkins’ MJs carotenoids are more bioaccessible after digestion. It is easy for carotenoids in ‘Tommy Atkins’ MJs fermented by L56 strains to be bio-transformed into carotenoid components during digestion. The increased bioaccessibility of carotenoids in this study supports the observation in LAB-fermented tropical leafy vegetables after digestion [31]. Furthermore, lactic acid fermentation of tomato pulp showed an increase in total carotenoids of 33.6 to 41.1%, and β-carotene content by up to 69% [35]. The bioaccessible carotenoids (β-carotene, lutein, and lycopene) and their release in the small intestine are vulnerable to absorption across the intestinal barrier and are affected by processing method, food matrix, dietary fat and fibre content, host-related factors, and interactions with other dietary components, type of carotenoids and its binding vehicles [32], hence, the fermentation of ‘Sabre’ MJs with L56 can ensure the delivery of quality and bioaccessible carotenoids to the body based on the concentration of bioaccessible β-carotene obtained in this study and can provide four times the RDA of carotenoids for adults.

### 3.4. Changes in Antioxidant Activities of MJs from Different Cultivars Fermented with LAB after In Vitro GI Digestion 

High importance is placed upon the post-digestion values for juices because the in vitro digestion model gives an indication of the bioavailable antioxidants in the juice [36]. The antioxidant power (FRAP) decreased in digested MJs across all cultivars and digestion phases and the decrease was highest in the DF (Table 3). The undigested MJs (UFU 0 h) had the highest FRAP activity across individual cultivars compared to the GF of fermented MJs. The FRAP decreased with the digestion of the MJs and L75-fermented ‘Sabre’ MJs had the highest antioxidant power (252.73 uM TEAC/mL) compared to L56 and L56 + 75-fermented and UFD MJs GF from all cultivars. However, the fold decrease in FRAP activity was not significantly different between L75 and L56 (1.42-fold) folds of ‘Peach’ and ‘Sabre’ but differed from ‘Tommy Atkins’ cultivar MJs (1.23-fold). The bioaccessible FRAP increased in fermented fractions and was highest in L75-fermented MJs GF in ‘Peach’ (70.60%), ‘Sabre’ (70.81%), and ‘Tommy Atkins’ (82.20%) compared to other fermented digests. The observed lowering FRAP activity after digestion could be related to the loss observed in the TPC content of the MJs digested fractions. Hence, the antioxidant power of LAB-fermented MJs remains mildly stable in GF after digestion, thus supporting the observation of Zhang et al. [37] in *Cinnamomum camphora* seed kernel after digestion.

Furthermore, a significant loss in FRAP activity was observed in the IF and DF MJs fractions, compared to the GF in all MJs from different cultivars. The antioxidant power of the IF fractions of the fermented MJs ranged from 96.73 to 152.51 uM TEAC/mL and was highest in ‘Sabre’ MJs fermented with L75 relative to other fermented MJs digests. The UFU MJs from different cultivars: ‘Peach’ (3.46-fold), ‘Sabre’ (2.87-fold), and ‘Tommy Atkins’ (3.08-fold) had the highest fold decrease in FRAP activity at the IF phase. Generally, IF digests of L75-fermented MJs across cultivars ‘Peach’ (2.48-fold, 40.36%), ‘Sabre’ (2.34-fold, 42.73%), and ‘Tommy Atkins’ (2.23-fold, 44.92%) had the lowest fold decrease in antioxidant power relative to the UFU MJs. Hence, LAB fermentation aided in the preservation of antioxidant power in all MJs. 

Likewise, the DF fractions of L75-fermented ‘Peach’, ‘Sabre’, and ‘Tommy Atkins’ MJs had the lowest fold decrease (6.01-fold, 5.30-fold, and 4.90-fold) in antioxidant power (16.64%, 18.88%, 20.40%), respectively, when compared to the UFD 24 h of respective cultivars. A similar reduction in antioxidant power was observed in beetroot juice during the duodenal and DF phases [38]. This is also observed in fermented papaya puree [14] and apple, orange, kiwi, pomelo, and grape juice after digestion [39]. There may be a reduction in antioxidant power due to the breakdown of bioactive compounds during digestion [40]. Other studies not limited to Wang et al. [41], however, have reported higher antioxidant power activities in fermented tomato juice after digestion and the differences observed may be due to the different types of phenolic compounds in food substrates. The higher fold decrease in antioxidant power of fermented MJs fractions at the duodenal phase compared to the GF phase could be related to the pH change from acidic to alkaline [37]. Additionally, a significant positive correlation (*p* < 0.01) was established with the antioxidant power (FRAP) (r = 0.92) as shown in (Table 4). The lower bioaccessibility of the antioxidant power at the GF, IF, and DF could be due to the partial use of some phenolic compounds present in the MJs during the continuous LAB activity during digestion relative to the UFD MJs, thus supporting the assertion of Selma et al. [23] and Valero-Cases et al. [38]. Similar results were reported in digested LAB-fermented papaya purees [14].

**Figure 3:**
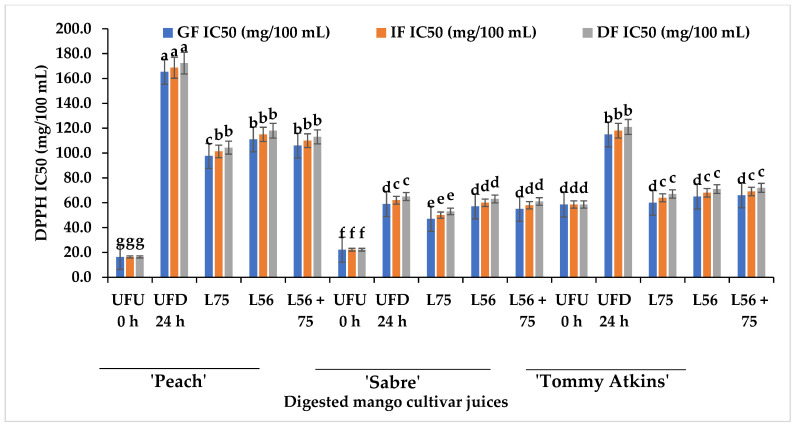
DPPH antioxidant scavenging activity of LAB-fermented MJs from different cultivars after in vitro GI digestion. Bars are mean ± standard deviation and different alphabets on similar colored bars indicate significant difference at *p* ≤ 0.05. Keys: *Ltp. plantarum* (*L75*); *Leu. pseudomesenteroides* (*L56*); *Leu. pseudomesenteroides 56* + *Ltp. plantarum 75* (*L56 + 75)*, gastric fraction (GF), Intestinal phase, Dialysis phase (DF); 2-diphenyl-1-picrylhydrazyl (DPPH); UFU 0 h—raw unfermented undigested Mango juice (control); UFD 24 h—unfermented digested MJs stored for 24 h.

The DPPH antioxidant scavenging activity of undigested (UFU 0 h), digested (UFD 24 h) and fermented MJs ranged from IC_50_ 16.33 mg/100 mL to IC_50_ 172.33 mg/100 mL and was highest in the UFU 0 h ‘Peach’ MJs and ‘Sabre’ UFD 24 h at GF (Figure 3). Compared to the LAB-fermented MJs from other cultivars, *L75*-fermented ‘Sabre’ MJs had the highest DPPH scavenging activity in the GF (IC_50_ 47 mg/100 mL). Furthermore, ‘Sabre’ MJs fermented with *L56* (IC_50_ 57 mg/100 mL) or *L56 + 75* (IC_50_ 55 mg/100 mL) demonstrated higher DPPH scavenging activity than ‘Peach’ and ‘Tommy Atkins’ MJs. 

**Figure 4:**
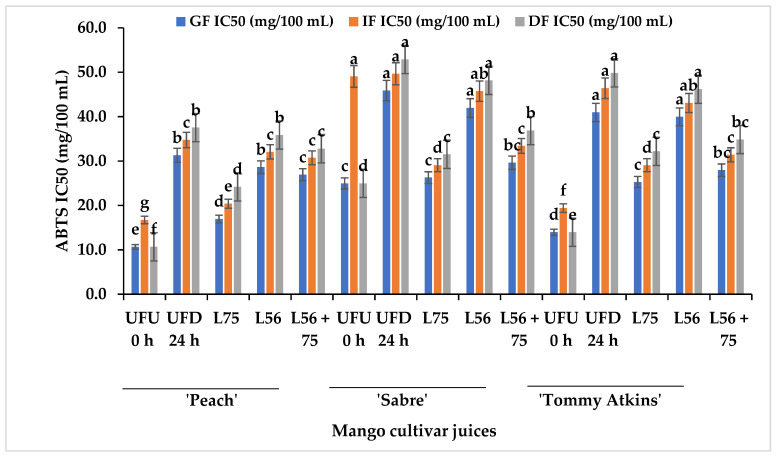
ABTS antioxidant scavenging activity of LAB-fermented MJs from different cultivars after in vitro GI digestion. Bars are mean ± standard deviation and different alphabets on similar colored bars indicate significant difference at *p* ≤ 0.05. Keys: *Ltp. plantarum* (L75); *Leu. pseudomesenteroides* (L56); *Leu. pseudomesenteroides 56* + *Ltp. plantarum 75* (L56 + 75), gastric fraction (GF), Intestinal phase, Dialysis phase (DF); 2.2-Azinobis-(3-ethyl-benzothiazoline-6- sulfonic acid (ABTS); UFU 0 h—raw unfermented undigested Mango juice (control); UFD 24 h—unfermented digested MJs stored for 24 h.

The consequence of reduced TPC shown in Table 1 could have influenced the loss in the DPPH scavenging abilities in the fractions. Figure 4 shows the effect of GI digestion on ABTS scavenging activity of fermented and UFU 0 h MJs from different cultivars. The ABTS scavenging activity ranged from IC_50_ 10.67 mg/100 mL to IC_50_ 45.88 mg/100 mL at the GF, IC_50_ 16.73–49.06 mg/100 mL at the IF and IC_50_ 10.67–52.88 mg/100 mL at the DF. Although the ABTS scavenging activity significantly decreased with the digestion of fermented and UFU MJs across digestion phases and cultivars. Similarly, ABTS activity decreased in vegetable juices after digestion [37]. Fermentation improved the ABTS antioxidant activity of fermented and digested ‘Peach’, ‘Sabre’, and ‘Tommy Atkins’ MJs compared to the unfermented (UFD 24 h). Regarding the effect of digestion on ABTS antioxidant activities of LAB-fermented MJs from different cultivars, *L75*-fermented ‘Peach’ MJs digesta had the highest ABTS antioxidant scavenging activity (IC_50_ 24.1 mg/100 mL) compared to MJs digesta from ‘Sabre’ and ‘Tommy Atkins’ cultivars at the dialysis phases. Furthermore, the low pH in the LAB-fermented MJs coupled with the condition at the GF and pepsin activity may have favoured the release of metabolites with high antioxidant activity in the digest [38]. Zhou et al. [25] reported an increase in ABTS activity of fermented kiwi fruit juices after digestion. The neutral pH, bile salts, and presence of pancreatic enzymes during the intestinal phase may have aided the biotransformation of some phenolic compounds in MJs fermenting by LAB strains thereby enhancing the preservation of ABTS antioxidant scavenging activity compared to the unfermented. A positive strong correlation exists between TPC and DPPH scavenging activity (r = 0.61) and ABTS scavenging activity (r = 0.68) as shown in Table 4. The changes in the antioxidant ability of fractions at the gastric or intestinal phases depend on the type of bioactive compounds in the food matrix [39]. In this study, we observed a higher DPPH activity in LAB-fermented fruit juices during the gastric phase, and a decrease during intestinal and dialysis phases. A reduction in DPPH activity during the intestinal and dialysis phases suggests that LAB-fermented MJs may act as functional foods to manage free radical generation in the body. The carotenoids and phenolic compounds in food have been reported to contribute significantly to the antioxidant properties of food, hence the positive correlation observed between TPC and FRAP, ABTS, and DPPH implies the significant contribution of TPC to the antioxidant activities in fermented mango juices. A similar report showing a strong correlation between the TPC and total antioxidant activities and a non-significant relationship with the TCC was reported in carrots [40]. 

Values represent the coefficient of correlations at *p* ≤ 0.05. DPPH (1,1-Diphenyl-2-picryl-hydrazyl); FRAP (Ferric Reducing Antioxidant Power); ABTS (2,2-Azinobis-(3-ethyl-benzothiazoline-6-sulfonic acid); TCC (β-carotene content); and TPC (Total phenol content).

## 4. Conclusions

In general, the digestion of LAB-fermented MJs results in the depletion of bioaccessible and bioavailable phenolic compounds. This invariably reduces the antioxidant activities of fermented MJs, as observed in this study. However, *L75*-fermented MJs preserved the phenolic contents and antioxidant properties in vitro during GI digestion while fermentation with *L56* preserved the carotenoid compounds in MJs. The type of mango cultivar and LAB strains influenced the absorption of TPC, carotenoid compounds, and antioxidant activities in digested MJs. Fermentation of MJs with L56 or L75 would enhance the bioaccessibility of β-carotene, zeaxanthin, lutein, trans-β-carotene, and antioxidants, respectively, in the gut. L56-fermented ‘Sabre’ MJs could deliver four times the RDA of carotenoids to the target sites in the body of adults. In contrast, L75 MJs fermented with ‘Peach’ could be effective to counteract oxidants generated in the body when consumed. The high biotransformation of carotenoids in *L56*-fermented MJs suggests that LAB utilized carotenoids for their metabolism, hence the *L56* could serve as probiotics in the gut. Digestion of fermented MJs had no significant reduction on the surviving LAB population, suggesting that LAB-fermented MJs may have the potential to function as probiotics in the gut in addition to their antioxidative properties. Further research could look into the effect of in vitro digestion of fermented MJs on individual phenolic compounds which would highlight the bioaccessible compounds in digested fermented MJs.

## Figures and Tables

**Table 1:** TPC of MJs from different cultivars fermented by LAB after in vitro GI digestion.

Mango Juices (MJs)	GF (mg/100 mL)	Fold Decrease	Bioacc %	IF (mg/100 mL)	Fold Decrease	Bioacc %	DF (mg/100 mL)	Fold Decrease	Bioacc %
‘Peach’									
UFU 0 h	1171.41 ± 0.57 a			1171.41 ± 0.57 a			1171.41 ± 0.57 a		
UFD 24 h	612.50 ± 0.57 k	1.91 ± 0.05 c	52.29	314.06 ± 0.1 n	3.73 ± 0.10 b	26.81	216.68 ± 0.53 n	5.41 ± 0.50 a	18.50
*L75*	822.17 ± 0.50 g	1.42 ± 0.05 h	70.19	511.45 ± 0.32 g	2.29 ± 0.31 i	43.66	366.21 ± 0.52 g	3.20 ± 0.50 i	31.26
*L56*	785.22 ± 0.56 i	1.49 ± 0.5 f	67.03	508.89 ± 0.18 h	2.30 ± 0.16 h	43.44	336.53 ± 0.55 i	3.48 ± 0.55 e	28.73
*L56 + 75*	821.80 ± 0.47 h	1.43 ± 0.15 g	70.15	452.15 ± 0.57 i	2.59 ± 0.57 g	38.60	342.58 ± 0.10 h	3.42 ± 0.15 f	29.25
‘Sabre’									
UFU 0 h	1167.03 ± 0.17 b			1167.03 ± 0.17 b			1167.03 ± 0.17 b		
UFD 24 h	690.95 ± 0.52 j	1.69 ± 0.05 e	59.21	387.02 ± 0.55 j	3.02 ± 0.55 c	33.16	226.56 ± 0.57 m	5.15 ± 0.53 c	19.41
*L75*	947.19 ± 0.50 c	1.23 ± 0.10 j	81.16	649.00 ± 0.15 d	1.80 ± 0.16 l	55.61	399.46 ± 0.57 d	2.92 ± 0.57 l	34.23
*L56*	882.84 ± 0.57 e	1.32 ± 0.15 i	75.65	584.74 ± 0.28 e	2.00 ± 0.25 k	50.10	379.57 ± 0.57 f	3.07 ± 0.55 j	32.52
*L56 + 75*	881.79 ± 0.55 f	1.32 ± 0.05 i	75.56	579.13 ± 0.81 f	2.02 ± 0.80 j	49.62	387.94 ± 0.57 e	3.01 ± 0.26 k	33.24
‘Tommy Atkins’									
UFU 0 h	923.06 ± 0.44 d			923.06 ± 0.44 c			923.06 ± 0.44 c		
UFD 24 h	372.19 ± 0.57 o	2.48 ± 0.05 a	40.32	241.77 ± 0.57 o	3.82 ± 0.57 a	26.19	172.95 ± 0.57 o	5.34 ± 0.40 b	18.74
*L75*	496.02 ± 0.27 l	1.86 ± 0.05 d	53.74	329.58 ± 3.56 k	2.80 ± 3.45 f	35.71	283.22 ± 0.57 j	3.26 ± 0.47 h	30.68
*L56*	479.51 ± 0.50 m	1.93 ± 0.15 b	51.95	318.79 ± 0.57 l	2.90 ± 0.57 e	34.54	270.83 ± 0.57 k	3.41 ± 0.85 g	29.34
*L56 + 75*	478.08 ± 0.35 n	1.93 ± 0.05 b	51.79	314.83 ± 0.13 m	2.93 ± 0.15 d	34.11	251.23 ± 1.15 l	3.67 ± 0.20 d	27.22
LSD	0.51 ***	0.15 ***		0.18 ***	0.31 ***		0.17 ***	0.53 ***	

Values are mean ± standard deviation and different alphabets indicate significant difference at *p* ≤ 0.05. Keys: *Ltp. plantarum* 75 (L75); *Leu. pseudomesenteroides* 56 (L56); *Leu. pseudomesenteroides* 56 + *Ltp. plantarum* 75 (L56 + 75); gastric fraction (GF); Intestinal fraction; dialysis fraction (DF); total phenolic content (TPC), UFU 0 h—raw unfermented and undigested MJs (Mango juice) (control); UFD 24 h—unfermented digested MJs stored for 24 h; Bioacc—bioaccessibility %—Percentage; Least significant difference (LSD); *** = *p* ≤ 0.05.

**Table 2:** Carotenoid profile of MJs from different cultivars fermented with LAB after in vitro GI digestion.

**Mango Cultivar Juices (MJs)**	**GF (mg/mL)**	**Fold Increase/Decrease**	**Bioacc (%)**	**IF (mg/mL)**	**Fold Decrease**	**Bioacc (%)**
**β-carotene concentration**						
‘Peach’						
UFU 0 h	61.20 ± 0.57 e			61.20 ± 0.57 a		
UFD 24 h	70.63 ± 0.37 d	1.15 ± 0.01 h	115.41	41.83 ± 0.57 e	−1.46 ± 0.01 b	68.35
L75	74.43 ± 0.40 c	1.21 ± 0.01 g	121.62	48.63 ± 0.57 c	−1.26 ± 0.01 g	79.46
L56	80.63 ± 0.30 a	1.32 ± 0.01 e	131.75	50.43 ± 0.57 b	−1.21 ± 0.01 h	82.40
L56 + 75	77.96 ± 0.22 b	1.27 ± 0.01 f	127.39	44.86 ± 0.57 d	−1.36 ± 0.01 e	73.30
‘Sabre’						
UFU 0 h	19.9 ± 0.57 l			19.9 ± 0.57 g		
UFD 24 h	28.63 ± 0.49 i	1.44 ± 0.01 d	143.87	14.83 ± 0.57 m	−1.34 ± 0.01 f	74.52
L75	34.73 ± 0.57 h	1.75 ± 0.01 c	174.52	18.07 ± 0.57 k	−1.10 ± 0.01 i	90.80
L56	51.53 ± 0.57 f	2.59 ± 0.01 a	258.94	19.7 ± 0.57 h	−1.01 ± 0.01 k	98.99
L56 + 75	48.73 ± 0.57 g	2.45 ± 0.01 b	244.87	18.8 ± 0.57 i	−1.06 ± 0.01 j	94.47
‘Tommy Atkins’						
UFU 0 h	20.2 ± 0.57 k			20.2 ± 0.57 f		
UFD 24 h	14.4 ± 0.1 o	−1.40 ± 0.01 m	71.29	12.63 ± 0.57 o	−1.60 ± 0.01 a	62.52
L75	16.83 ± 0.57 m	−1.20 ± 0.01 k	83.32	14.54 ± 0.57 m	−1.39 ± 0.01 d	71.98
L56	25.83 ± 0.57 j	1.27 ± 0.01 i	127.87	18.35 ± 0.57 j	−1.10 ± 0.01 i	90.84
L56 + 75	15.83 ± 0.57 n	−1.28 ± 0.01 l	78.37	14.11 ± 0.57 n	−1.43 ± 0.01 c	69.85
LSD	0.12 **	0.11 **		0.16 **	0.05 **	
**Trans-β-carotene concentration**						
**Mango juice**	**GF (µg/mL)**	**Fold increase**	**Bioacc (%)**	**IF (µg/mL)**	**Fold increase/decrease**	**Bioacc (%)**
*‘Peach’*						
UFU 0 h	5.27 ± 0.05 f			5.26 ± 0.17 b		
UFD 24 h	6.03 ± 0.1 e	1.14 ± 0.11 f	114.42	4.00 ± 0.13 f	−1.31 ± 0.13 e	76.4
L75	7.23 ± 0.05 b	1.37 ± 0.05 e	137.19	4.23 ± 0.15 d	−1.24 ± 0.15 d	75.54
L56	9.17 ± 0.25 a	1.74 ± 0.21 b	174.00	7.2 ± 0.27 a	1.37 ± 0.11 a	136.88
L56 + 75	6.30 ± 0.51 c	1.19 ± 0.45 f	119.54	4.3 ± 0.19 d	1.22 ± 0.11 b	81.74
*‘Sabre’*						
UFU 0 h	4.03 ± 0.05 i			4.03 ± 0.19 f		
UFD 24 h	4.27 ± 0.12 h	1.05 ± 0.30 g	105.95	2.26 ± 0.23 h	−1.78 ± 0.21 f	56.07
L75	5.27 ± 0.23 f	1.30 ± 0.31 e	130.76	3.2 ± 0.27 g	−1.25 ± 0.22 d	79.4
L56	7.20 ± 0.55 b	1.78 ± 0.42 b	178.66	4.4 ± 0.57 c	1.09 ± 0.16 c	109.18
L56 + 75	6.23 ± 0.45 cd	1.54 ± 0.18 c	154.59	4.23 ± 0.15 d	1.04 ± 0.20 c	104.96
‘Tommy Atkins’						
UFU 0 h	3.03 ± 0.03 K			3.03 ± 0.07 g		
UFD 24 h	3.27 ± 0.07 J	1.07 ± 0.41 g	107.92	1.26 ± 0.24 i	−2.40 ± 0.32 g	41.58
L75	4.27 ± 0.43 h	1.4 ± 0.22 d	140.92	2.26 ± 0.30 h	−1.34 ± 0.33 e	74.58
L56	6.17 ± 0.11 d	2.03 ± 0.53 a	203.63	4.16 ± 0.48 e	1.37 ± 0.21 a	137.29
L56 + 75	5.17 ± 0.75 g	1.70 ± 0.48 b	170.62	3.16 ± 0.03 g	1.04 ± 0.11 c	104.29
**LSD**	0.01 **	0.01 **	0.03 **	0.02 **	0.04 **	0.08 **
**Zeaxanthin concentrations**						
**Mango juice**	**GF (µg/mL)**	**Fold increase**	**Bioacc (%)**	**GF (µg/mL)**	**Fold increase/decrease**	**Bioacc (%)**
‘Peach’						
UFU 0 h	4.26 ± 0.25 e			4.26 ± 0.25 b		
UFD 24 h	6.23 ± 0.35 b	1.46 ± 0.57 e	146.2	4.23 ± 0.23 b	−1.00 ± 0.25 e	99.29
L75	5.03 ± 0.55 d	1.18 ± 0.21 h	118.07	3.03 ± 0.53 d	−1.41 ± 0.53 f	71.12
L56	8.16 ± 0.25 a	1.91 ± 0.09 b	191.54	6.16 ± 0.16 a	1.44 ± 0.15 b	144.60
L56 + 75	5.3 ± 0.32 c	1.24 ± 0.34 g	124.41	3.3 ± 0.30 c	−1.29 ± 0.35 f	77.46
‘Sabre’						
UFU 0 h	3.13 ± 0.51 g			3.13 ± 0.51 c		
UFD 24 h	3.43 ± 0.33 f	1.09 ± 0.11 i	109.58	1.43 ± 0.35 g	−2.18 ± 0.35 h	45.68
L75	4.26 ± 0.75 e	1.36 ± 0.43 f	136.10	2.26 ± 0.22 e	−1.38 ± 0.22 f	72.20
L56	6.23 ± 0.09 b	1.99 ± 0.19 a	199.04	4.23 ± 0.22 b	1.35 ± 0.23 c	135.14
L56 + 75	5.23 ± 0.32 c	1.67 ± 0.27 c	167.09	3.23 ± 0.37 c	1.03 ± 0.38 d	103.19
‘Tommy Atkins’						
UFU 0 h	2.06 ± 0.05 i			2.06 ± 0.05 f		
UFD 24 h	2.26 ± 0.83 h	1.09 ± 0.21 i	109.71	0.26 ± 0.09 i	−7.92 ± 0.09 i	12.62
L75	3.2 ± 0.52 g	1.55 ± 0.60 d	155.33	1.2 ± 0.23 h	−1.71 ± 0.24 g	58.25
L56	5.16 ± 0.77 c	2.50 ± 0.17 a	250.48	3.16 ± 0.65 c	1.53 ± 0.55 a	153.39
L56 + 75	4.16 ± 0.06 e	2.01 ± 0.14 a	201.94	2.16 ± 0.15 f	1.04 ± 0.16 d	104.85
**LSD**	0.08 **	0.05 **	0.02 **	0.02 **	0.01 **	0.03 **
**Lutein concentration**						
**Mango juice**	**GF (µg/mL)**	**Fold decrease**	**Bioacc (%)**	**IF (µg/mL)**	**Fold increase/ decrease**	**Bioacc (%)**
‘Peach’						
UFU 0 h	3.93 ± 0.15 d			3.93 ± 0.15 b		
UFD 24 h	5.23 ± 0.23 b	1.33 ± 0.25 e	133.03	3.23 ± 0.20 b	−1.21 ± 0.20 d	82.18
L75	5.03 ± 0.09 b	1.27 ± 0.10 f	127.98	3.03 ± 0.05 b	−1.29 ± 0.05 d	77.09
L56	6.5 ± 0.63 a	1.65 ± 0.09 b	165.39	6.16 ± 0.18 a	1.56 ± 0.20 a	156.74
L56 + 75	4.63 ± 0.36 c	1.17 ± 0.35 g	117.81	2.63 ± 0.36 c	−1.49 ± 0.38 d	66.92
‘Sabre’						
UFU 0 h	2.8 ± 0.25 e			2.8 ± 0.25 c		
UFD 24 h	3.1 ± 0.31 d	1.10 ± 0.31 h	110.71	0.76 ± 0.63	−3.68 ± 0.60 e	27.14
L75	3.93 ± 0.35 d	1.40 ± 0.33 d	140.35	1.93 ± 0.33 d	−1.45 ± 0.33 d	68.92
L56	5.23 ± 0.55 b	1.86 ± 0.53 a	186.78	3.23 ± 0.28 b	1.15 ± 0.29 b	115.35
L56 + 75	4.56 ± 0.65 c	1.62 ± 0.55 b	162.85	2.9 ± 0.28 c	1.03 ± 0.28 c	103.57
‘Tommy Atkins’						
UFU 0 h	2.4 ± 0.19 e			2.4 ± 0.19 c		
UFD 24 h	2.6 ± 0.20 e	1.08 ± 0.25 i	108.33	0.6 ± 0.35 e	−4.00 ± 0.36 f	25.00
L75	2.8 ± 0.82 e	1.16 ± 0.85 h	116.66	0.53 ± 0.30 e	-4.52 ± 0.30 g	22.08
L56	4.5 ± 0.95 c	1.87 ± 0.95 a	187.5	2.5 ± 0.25 c	1.04 ± 0.24 c	104.16
L56 + 75	3.83 ± 0.33 d	1.59 ± 0.32 c	159.58	1.83 ± 0.30 d	−1.31 ± 0.30 d	76.25
LSD	0.01 **	0.04 **	0.44 **	0.01 **	0.01 **	0.03 **
**Alpha carotene concentration**						
**Mango juice**	**GF (µg/mL)**	**Fold increase**	**Bioacc (%)**	**IF (µg/mL)**	**Fold increase/decrease**	**Bioacc (%)**
‘Peach’						
UFU 0 h	4.93 ± 0.35 d			4.93 ± 0.05 b		
UFD 24 h	5.36 ± 0.13 c	1.08 ± 0.15 g	108.72	4.23 ± 0.30 b	−1.16 ± 0.30 c	85.80
L75	5.63 ± 0.65 c	1.14 ± 0.64 f	114.19	3.36 ± 0.22 c	−1.46 ± 0.20 d	68.15
L56	7.5 ± 0.55 a	1.52 ± 0.56 b	152.12	5.5 ± 0.15 a	1.11 ± 0.15 a	111.56
L56 + 75	6.23 ± 0.13 b	1.26 ± 0.14 e	126.36	2.96 ± 0.23 d	−1.66 ± 0.25 d	60.04
‘Sabre’						
UFU 0 h	4.03 ± 0.05 d			4.03 ± 0.05 b		
UFD 24 h	3.93 ± 0.33 e	−1.02 ± 0.30 h	95.51	2.26 ± 0.22 d	−1.78 ± 0.24 d	56.07
L75	4.93 ± 0.15 d	1.22 ± 0.15 e	122.33	2.93 ± 0.13 d	−1.37 ± 0.12 cd	72.70
L56	6.2 ± 0.27 b	1.53 ± 0.27 b	153.84	4.56 ± 0.25 b	1.13 ± 0.25 a	113.15
L56 + 75	5.56 ± 0.45 c	1.37 ± 0.46 d	137.96	4.2 ± 0.12 b	1.04 ± 0.12 b	104.21
‘Tommy Atkins’						
UFU 0 h	3.36 ± 0.25 e			3.36 ± 0.25 c		
UFD 24 h	3.6 ± 0.09 e	1.07 ± 0.10 g	107.14	1.26 ± 0.15 e	−2.66 ± 0.16 e	37.5
L75	3.93 ± 0.45 e	1.16 ± 0.50 f	116.96	nd	nd	nd
L56	5.5 ± 0.15 c	1.63 ± 0.14 a	163.69	3.5 ± 0.45 c	1.04 ± 0.28 b	104.16
L56 + 75	4.83 ± 0.12 d	1.43 ± 0.12 c	143.75	nd	nd	nd
LSD	0.06 *	0.02 **	0.01 **	0.05 **	0.02 **	

Values are mean ± standard deviation and different alphabets indicate significant difference at *p* ≤ 0.05. Keys: *Ltp. plantarum* (*L75*); *Leu. pseudomesenteroides* (*L56*); *Leu. pseudomesenteroides* 56 + *Ltp. plantarum* 75 (L56 + 75); gastric fraction (GF); Intestinal fraction; UFU 0 h—raw unfermented and undigested MJs (Mango juice) (control); UFD 24 h—unfermented digested MJs stored for 24 h; Bioacc—bioaccessibility; nd—not detected; %—Percentage; Least significant difference (LSD). * = *p* ≤ 0.05; ** = *p* ≤ 0.005.

**Table 3:** Antioxidant power (FRAP) of MJs of different cultivars fermented with LAB after in vitro GI digestion.

Mango Juices	GF (uM TEAC/mL)	Fold Decrease	Bioacc %	IF (uM TEAC/mL)	Fold Decrease	Bioacc %	DF (uM TEAC/mL)	Fold Decrease	Bioacc %
‘Peach’									
UFU 0 h	354.19 ± 5.74 b			354.19 ± 5.74 b			354.19 ± 5.74 a		
UFD 24 h	235.17 ± 3.01 f	−1.51 ± 0.31 c	66.40	102.51 ± 42.63 f	−3.46 ± 0.19 d	28.94	55.84 ± 2.30 d	−6.34 ± 0.30 d	15.77
*L75*	250.06 ± 7.00 d	−1.42 ± 0.06 b	70.60	142.95 ± 17.05 de	−2.48 ± 0.05 b	40.36	58.95 ± 3.67 cd	−6.01 ± 0.50 c	16.64
*L56*	249.4 ± 14.24 d	−1.42 ± 0.04 b	70.41	126.4 ± 0.57 e	−2.80 ± 0.70 c	35.69	56.95 ± 2.14 d	−6.22 ± 0.15 d	16.08
*L56+75*	238.73 ± 9.43 f	−1.48 ± 0.23 c	67.40	137.4 ± 4.16 de	−2.58 ± 0.16 b	38.79	55.84 ± 2.90 d	−6.34 ± 0.20 d	15.77
‘Sabre’									
UFU 0 h	356.91 ± 7.42 a			356.91 ± 10.33 a			356.91 ± 0.76 a		
UFD 24 h	238.06 ± 4.07 f	−1.50 ± 0.07 c	66.70	124.28 ± 10.02 e	−2.87 ± 0.22 c	34.82	54.95 ± 3.00 d	−6.50 ± 0.58 e	15.40
*L75*	252.73 ± 30.30 d	−1.41 ± 0.40 b	70.81	152.51 ± 28.83 d	−2.34 ± 0.08 a	42.73	67.4 ± 3.28 c	−5.30 ± 0.25 b	18.88
*L56*	251.17 ± 31.67 d	−1.42 ± 0.61 b	70.37	127.17 ± 2.34 e	−2.81 ± 0.41 c	35.63	65.4 ± 4.72 c	−5.46 ± 0.20 c	18.32
*L56 + 75*	247.84 ± 3.01 de	−1.44 ± 0.13 b	69.44	139.4 ± 11.37 de	−2.56 ± 0.13 b	39.06	63.62 ± 1.53 c	−5.61 ± 0.06 c	17.83
‘Tommy Atkins’									
UFU 0 h	297.74 ± 48.07 c			297.74 ± 48.07 c			297.74 ± 48.07 b		
UFD 24 h	197.4 ± 44.77 g	−1.51 ± 0.17 c	66.30	96.73 ± 34.11 f	−3.08 ± 0.11 d	32.49	41.4 ± 1.38 e	−7.19 ± 0.33 f	13.90
*L75*	244.73 ± 8.00 e	−1.22 ± 0.33 a	82.20	133.73 ± 0.57 e	−2.23 ± 0.51 a	44.92	60.73 ± 8.44 c	−4.90 ± 0.24 a	20.40
*L56*	241.4 ± 8.22 e	−1.23 ± 0.28 a	81.08	104.73 ± 34.66 f	−2.84 ± 0.33 c	35.17	46.95 ± 10.79 e	−6.34 ± 0.68 d	15.77
*L56 + 75*	241.4 ± 6.93 e	−1.23 ± 0.31 a	81.08	127.84 ± 2.1 e	−2.33 ± 0.12 a	42.94	44.95 ± 1.53 e	−6.62 ± 0.33 e	15.10
LSD	3.46 **	0.01 **		3.48 **	0.22 **		3.49 **	0.33 **	

Values are mean ± standard deviation and different alphabets indicate significant difference at *p* < 0.05. Keys: *Ltp. Plantarum* 75 (L75); *Leu. pseudomesenteroides* 56 (L56); *Leu. pseudomesenteroides* 56 + *Ltp. plantarum* 75 (L56 + 75), gastric fraction (GF), Intestinal phase, Dialysis phase (DF), Ferric reducing antioxidant power (FRAP); UFU 0 h—raw unfermented undigested MJs (Mango juice) (control); UFD 24 h—unfermented digested MJs stored for 24 h; Trolox equivalent antioxidant capacity (TEAC); Bioacc—bioaccessibility; %—Percentage; Least significant difference (LSD); ** = *p* ≤ 0.005.

**Table 4:** Pearson correlation coefficients (r) of antioxidants and their activities in LAB strains fermented MJs from different cultivars after in vitro GI digestion.

	TPC	TCC	FRAP	ABTS	DPPH
TPC	1				
TCC	0.41	1			
FRAP	0.92	0.44	1		
ABTS	0.68	−0.31	−0.56	1	
DPPH	0.61	0.15	−0.52	0.23	1

## Data Availability

All data included in the Manuscript and Supplementary tables are attached here.

## Supplementary Materials

The following supporting information can be downloaded at: https://www.mdpi.com/article/10.3390/foods11172702/s1, Table S1: Quantification and Validation of the carotenoids.
