# Supplementary material for: The Bioaccessibility and Antioxidant Activities of Fermented Mango Cultivar Juices after Simulated In Vitro Digestion
Source: Foods. 2022 Sep 5;11(17):2702. doi: 10.3390/foods11172702 (PMC9455754; doi:10.3390/foods11172702)
Supplement: Supplementary file 1 [file foods-11-02702-s001.zip › foods-1850654-supplementary.pdf]

Supplementary data

Table S1: Quantification and Validation of the carotenoids

Carotenoid	Equation	Regression coefficient (R^2)	LOD	LOQ
B-carotene	Y=140594x + 36337	0,9979	6,32	19,16
Trans β-carotene	Y=30127x + 15	0,9781	18,02	54,61
α-carotene	Y=26242x-47686	0,9987	4,39	13,29
Lutein	Y=94894x + 834002	0,9538	26,52	80,37
Zeaxanthin	Y=68769x-740682	0,9787	7,31	22,15
